# Combination of Ethoxybenzyl‐Diethylenetriamine Pentaacetic Acid‐Enhanced Magnetic Resonance Imaging and a Serum Biomarker Is Useful in the Diagnosis of Hepatic Sinusoidal Disorder After Chemotherapy Treatment

**DOI:** 10.1002/ags3.70092

**Published:** 2025-09-09

**Authors:** Tomonari Shimagaki, Keishi Sugimachi, Takahiro Tomino, Takeshi Kurihara, Emi Onishi, Yutaro Shimomura, Kenji Shinozaki, Masaru Morita

**Affiliations:** ^1^ Department of Hepatobiliary and Pancreatic Surgery NHO Kyushu Cancer Center Fukuoka Japan; ^2^ Department of Diagnostic Imaging and Nuclear Medicine NHO Kyushu Cancer Center Fukuoka Japan; ^3^ Department of Gastroenterological Surgery NHO Kyushu Cancer Center Fukuoka Japan

**Keywords:** aspartate aminotransferase to platelet ratio index, blue liver, colorectal liver metastasis, gadolinium ethoxybenzyl‐diethylenetriamine pentaacetic acid‐enhanced magnetic resonance imaging, oxaliplatin

## Abstract

**Aim:**

Sinusoidal obstruction syndrome (SOS), also known as “blue liver (BL),” is a common hepatic injury following oxaliplatin‐based chemotherapy in patients with colorectal liver metastases (CRLM). Early non‐invasive identification of SOS is essential for safe hepatic resection and improved outcomes; however, this remains clinically challenging.

**Methods:**

We retrospectively analyzed 155 patients who underwent preoperative ethoxybenzyl‐diethylenetriamine pentaacetic acid‐enhanced magnetic resonance imaging (EOB‐MRI) and hepatic resection for CRLM between 2014 and 2022. Radiologists evaluated SOS on EOB‐MRI using a five‐point reticular signal grading scale. Aspartate aminotransferase to platelet ratio index (APRI) scores were calculated preoperatively. BL was confirmed intraoperatively based on characteristic liver discoloration. Correlations between EOB‐MRI scores, APRI, and clinical outcomes were analyzed using receiver operating characteristic curves, survival analysis, and multivariate statistics.

**Results:**

Of 70 patients treated with preoperative oxaliplatin, 25 (35.7%) exhibited intraoperative BL. Overall survival was significantly worse in the blue liver group (*p* = 0.0338), although disease‐free survival did not differ significantly. Patients in the BL group had significantly higher APRI and EOB‐MRI scores (*p* = 0.0028 and *p* < 0.0001, respectively). The combined EOB‐MRI assessment and APRI had the highest diagnostic ability for BL detection, yielding an area under the curve of 0.806, with 78.0% sensitivity and 67.7% specificity. Patients with high scores in both modalities exhibited significantly poorer overall survival.

**Conclusion:**

A combination of EOB‐MRI and APRI is a valuable non‐invasive tool for preoperative detection of SOS in patients with CRLM. This approach improves diagnostic accuracy, facilitates surgical planning, and may predict long‐term prognosis following hepatic resection.

## Graphical abstract

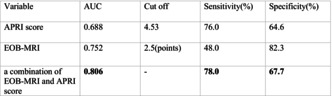

**Abbreviations**
ALT: alanine aminotransferase
APRI: Aspartate Aminotransferase to Platelet Ratio Index
AST: aspartate aminotransferase
BL: blue liver
CEA: carcinoembryonic antigen
CI: confidence interval
CRLM: colorectal cancer liver metastases
CT: computed tomography
DFS: disease‐free survival
EOB‐MRI: gadolinium‐ethoxybenzyl‐diethylenetriamine pentaacetic acid‐enhanced magnetic resonance imaging
HR: hazard ratio
ICG: indocyanine green
OS: overall survival
RLS: reticular low signal
ROC: receiver operating characteristic
SOS: sinusoidal obstruction syndrome
TBS: tumor burden score

## 1 Introduction

Colorectal cancer is a leading cause of cancer‐related morbidity and mortality worldwide, and hepatic metastases frequently occur in patients with advanced‐stage disease [1]. Surgical resection remains the mainstay treatment for colorectal cancer liver metastases (CRLM) when feasible. However, systemic chemotherapy, particularly oxaliplatin‐based regimens, is commonly administered before surgical intervention to downstage tumors and improve resectability [2]. Despite its efficacy, oxaliplatin is associated with hepatic sinusoidal obstruction syndrome (SOS), colloquially referred to as “blue liver” (BL), which can lead to portal hypertension and postoperative complications [3, 4].

An early and accurate diagnosis of SOS is crucial for optimizing patient management and improving surgical outcomes [5]. Aspartate aminotransferase to platelet ratio index (APRI) is a useful biomarker for detecting SOS preoperatively and predicting postoperative prognosis following hepatic resection for CRLM [6]. Imaging modalities, such as gadolinium‐ethoxybenzyl‐diethylenetriamine pentaacetic acid (Gd‐EOB‐DTPA)‐enhanced magnetic resonance imaging (EOB‐MRI), have also been investigated for their ability to detect SOS [7]. Gd‐EOB‐DTPA is a contrast agent that is taken up by liver cells and excreted in bile. In normal liver cells, contrast agents are absorbed, and the signal intensity increases; however, in SOS, liver cell function is impaired or damaged, resulting in reduced absorption and areas of decreased signal intensity. Therefore, the hepatocellular phase of EOB‐MRI can show a low reticular signal pattern suggestive of SOS, although its diagnostic accuracy remains debated [8, 9].

This study evaluated the usefulness of EOB‐MRI in diagnosing BL, the relationship between imaging findings suggestive of SOS on EOB‐MRI and APRI scores, and postoperative prognosis in patients undergoing hepatic resection for CRLM. A retrospective comparative analysis was conducted to determine whether preoperative imaging features correlated with SOS diagnosis and predicted long‐term survival outcomes.

## 2 Methods

### 2.1 Study Design

**FIGURE 1:**
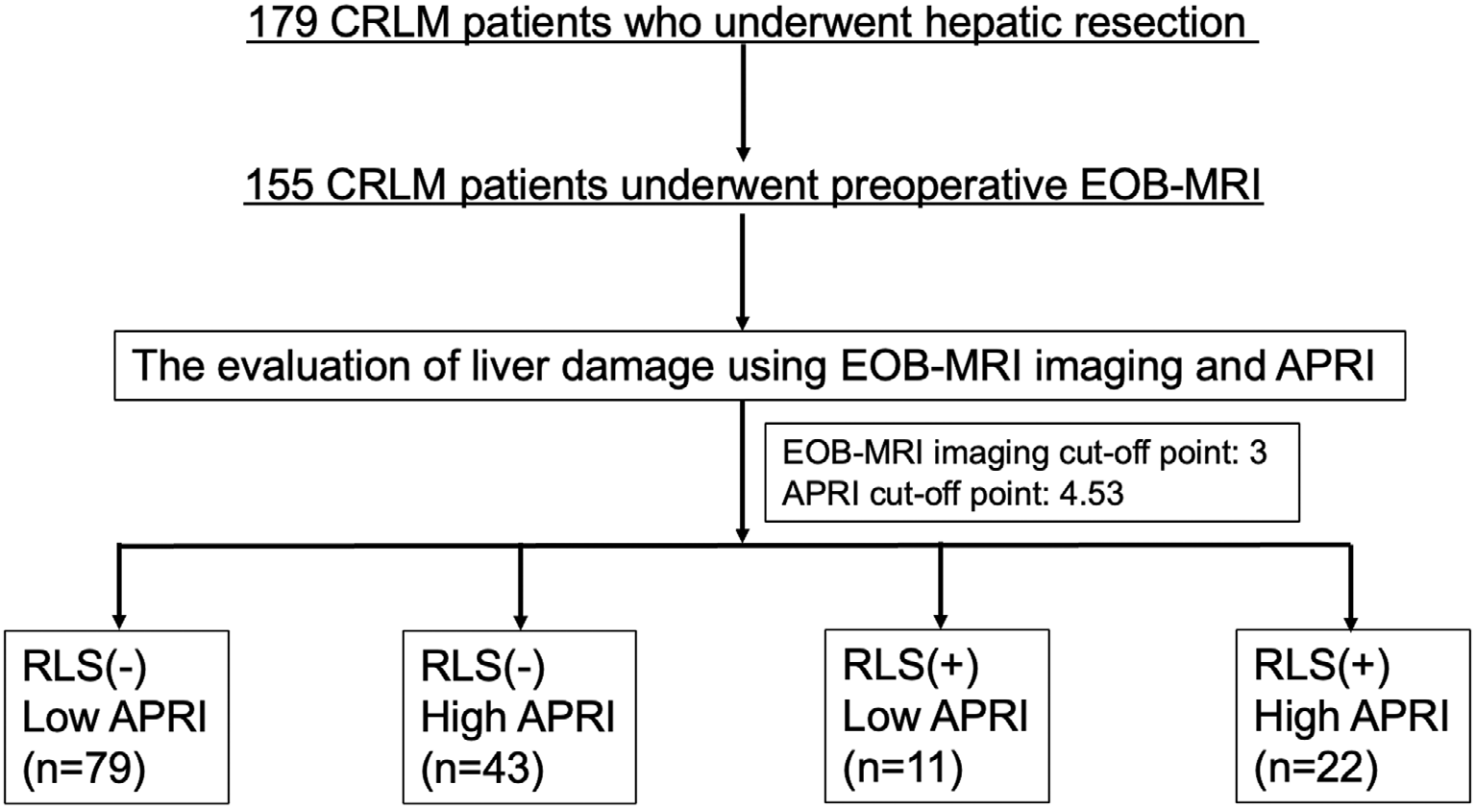
Flowchart of the study. CRLM, colorectal cancer liver metastases; EOB‐MRI, ethoxybenzyl‐diethylenetriamine pentaacetic acid‐enhanced magnetic resonance imaging; APRI, Aspartate Aminotransferase to Platelet Ratio Index; RLS, reticular low signal.

Medical records of 179 Japanese patients who met the following inclusion criteria were used in the present study: treatment with radical surgery for CRLM between January 2014 and June 2022, initial diagnosis of CRLM, and histological diagnosis of CRLM. The patients had no organ metastases other than liver metastases preoperatively. Among the 179 patients, we included 155 who underwent preoperative EOB‐MRI (Figure 1).

Resected hepatic specimens were reviewed by an independent pathologist. No evidence of cancer cell infiltration was identified at the surgical margins. Postoperatively, patients were monitored in the hospital at 3‐month intervals. During these follow‐up visits, the patients underwent measurement of liver function tests, carcinoembryonic antigen (CEA) levels, carbohydrate antigen 19‐9 levels, and additional blood analyses; additionally, patients underwent contrast‐enhanced computed tomography (CT) and/or MRI.

The treatment strategy for each patient was determined by a multidisciplinary Cancer Board, consisting of medical oncologists, gastrointestinal surgeons, and hepatic surgeons. Treatment plans were discussed at the time of the patient's initial consultation and upon the achievement of significant therapeutic responses. For patients with resectable CRLM and primary tumors, surgical resection followed by chemotherapy was performed in accordance with institutional protocols. The criteria for categorizing cases as clearly unresectable, suboptimally resectable, or unsuitable for curative resection were as follows: (i) synchronous metastases involving multiple organs; (ii) anticipated insufficient remnant liver volume or function after hepatectomy; (iii) CRLM invasion into critical hepatic structures, including the hilum and hepatic vein root, rendering complete resection without residual disease infeasible; and (iv) classification as Category H2 or H3 under the Japanese Classification of Colorectal Cancer (defined as > 5 hepatic metastatic lesions and/or maximum diameter > 5 cm), which is associated with a less favorable prognosis following hepatectomy compared with Category H1. Patients meeting these criteria were treated with chemotherapy, and the specific regimen was primarily selected by the oncologist according to the patient's overall condition [10].

We performed a retrospective comparative study of patients with CRLM who underwent hepatectomy to determine whether diagnostic imaging using EOB‐MRI is related to SOS and APRI, and if so, to life expectancy.

### 2.2 Definitions

Calistri et al. reported that BL referred to parenchymal venous congestion resulting from blockage of blood outflow, macroscopically characterized by an intraoperative subcapsular livid appearance and a similar “marble” bluish‐red discoloration on the cut surface [11]. Therefore, ≥ 3 surgeons determined the evaluation of oxaliplatin‐associated BL by confirming the color of the liver during the surgery [12, 13]. There was little variation among surgeons. The BL findings are shown in Figure S1.

In patients with multiple nodules, the size of the largest lesion was defined as the tumor size. Major hepatectomy was defined as resection of ≥ 3 Couinaud segments [14]. The tumor burden score (TBS) was defined as the distance from the origin of a Cartesian plane and comprised two variables [15]: the maximum tumor size (*x*‐axis) and number of tumors (*y*‐axis); thus, TBS^2^ = (maximum tumor diameter)^2^ + (number of tumors)^2^. For each patient, the maximum tumor diameter and number of tumors were obtained from the pathological report.

The following biological parameters were assessed preoperatively: platelet count, serum creatinine, serum aspartate aminotransferase (AST), serum alanine aminotransferase (ALT), gamma‐glutamyl transferase, alkaline phosphatase, serum total bilirubin, and prothrombin time. Finally, the APRI [16, 17] was calculated to determine its correlation with the pathologic findings. The formulas used for these calculations are as shown below. APRI could be influenced by hepatic or hematologic comorbidities. In our cohort, patients with autoimmune hepatitis, primary biliary cholangitis, hematologic disorders, or known hypersplenism unrelated to chemotherapy were not included. When the liver remnant was insufficient to ensure satisfactory postoperative liver function, preoperative liver volumetry was performed using three‐dimensional CT to ensure a safe hepatectomy procedure.

$$\begin{array}{c} \text{APRI}=\left(\mathrm{AST}\;\left[U/L\right]/\text{upper limit of the reference range}\right)\\ \times 100/\text{platelet count}\;\left(10^{9}/L\right) \end{array}$$

### 2.3 Preoperative Evaluation of SOS on EOB‐MRI by Radiologists

**FIGURE 2:**
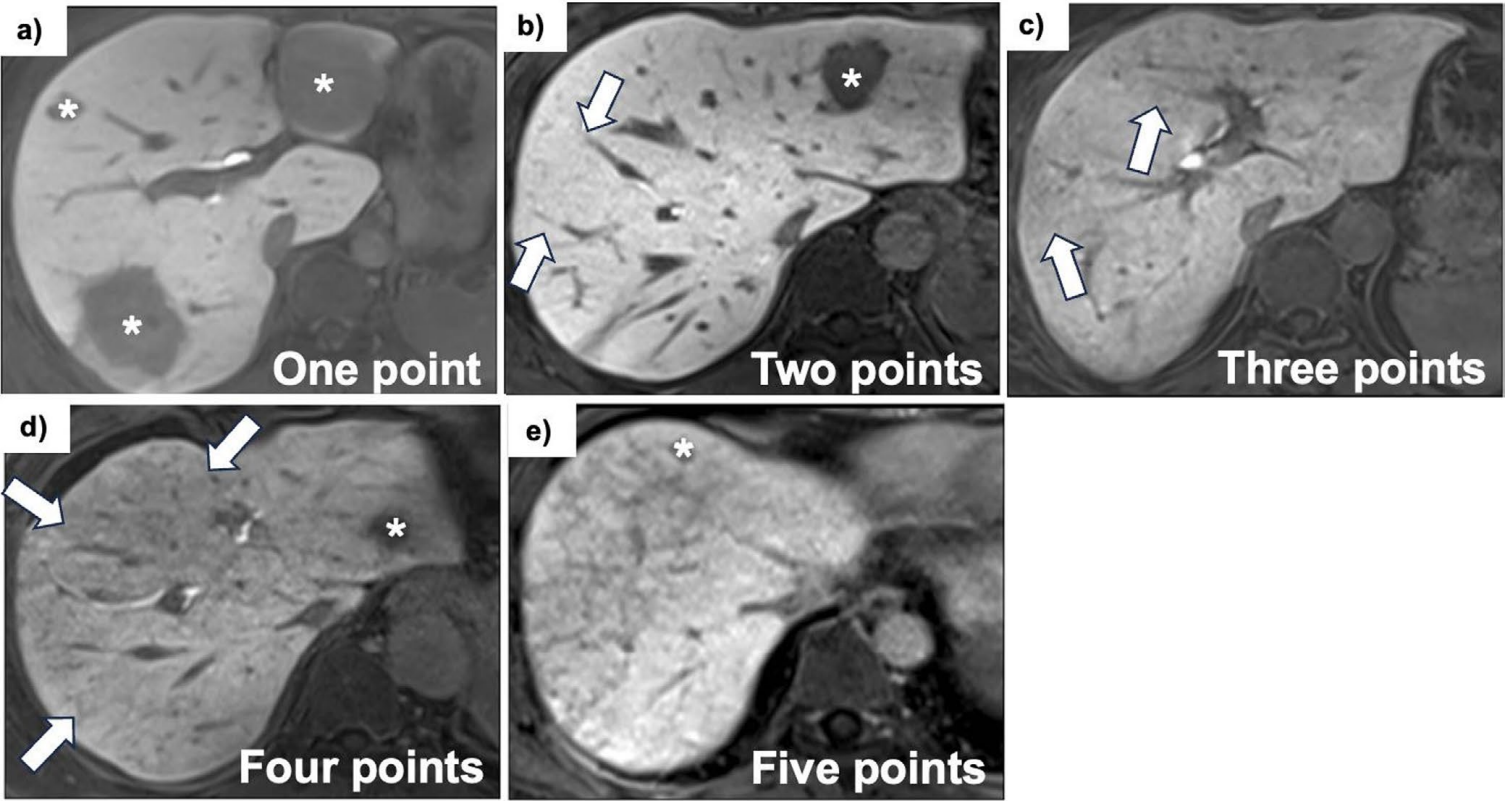
Representative hepatobiliary‐phase images from EOB‐MRI demonstrating the range of SOS severity, classified according to the degree of reticular hypointensity and the corresponding confidence levels: (a) Homogeneous high signal intensity across the liver parenchyma, indicating absence of SOS (confidence level = 1); (b) Faint reticular hypointensity (arrows) detectable in a limited number of sections, but the findings remain equivocal (confidence level = 2); (c) Fine reticular hypointensity localized to restricted areas (arrows), suggestive of mild SOS (confidence level = 3); (d) More pronounced reticulations distributed diffusely throughout all sections (arrows), consistent with moderate SOS (confidence level = 4); (e) Marked and extensive reticular hypointensity throughout the liver parenchyma, indicative of severe SOS (confidence level = 5). Hypointense nodular lesions indicated by asterisks (*) correspond to metastatic tumors, clearly distinguishable from SOS‐related alterations. EOB‐MRI, ethoxybenzyl‐diethylenetriamine pentaacetic acid‐enhanced magnetic resonance imaging; SOS, sinusoidal obstruction syndrome.

Two radiologists evaluated the presence of reticular low signal (RLS) in the hepatocellular phase on EOB‐MRI, suggestive of SOS on a five‐point scale from 1 to 5 (1 = definitely not present; 2 = probably not present; 3 = equivocal; 4 = probably present; 5 = definitely present) for image evaluation of SOS [18] (Figure 2). The EOB‐MRI evaluation was calculated based on the average score provided by the two radiologists.

### 2.4 Statistical Analysis

Clinicopathological records of all 179 patients were collected and retrospectively reviewed. Differences between groups (BL vs. non‐BL) were assessed using the Mann–Whitney *U* test. Comparisons of the same patient were performed using the paired Student's *t*‐test. Associations between variables were determined using the Fisher's exact or *χ* ^2^ test. Diagnostic performance of potential biomarkers was assessed using receiver operating characteristic (ROC) curves.

Disease‐free survival (DFS) and overall survival (OS) curves were calculated using the Kaplan–Meier method, and differences between the groups were assessed using the log‐rank test. Univariate and multivariate logistic regression analyses were performed to identify the independent determinants of OS. Statistical analyses were performed using JMP Pro software (ver. 15.1, SAS Institute, Cary, NC, USA). Statistical significance was set at a *p* value of < 0.05.

## 3 Results

### 3.1 Patient Characteristics

Patient characteristics are summarized in Table S1. The male to female ratio was 79:76, with a mean age of 64.5 years (range, 36–84 years). The mean (± standard deviation) body mass index prior to hepatectomy was 23.0 ± 0.3 kg/m^2^, and the mean serum albumin concentration was 4.0 ± 0.1 mg/dL. The primary tumor was located in the right colon (proximal to the splenic flexure) in 37 patients, in the left colon (distal to the splenic flexure) in 67 patients, and in the rectum in 51 patients. The mean preoperative serum concentrations of CEA and carbohydrate antigen 19‐9 were 6.6 ng/mL and 15.0 ng/mL, respectively. With regard to the distribution of CRLM, 95 patients had unilobar and 60 had bilobar involvement. In terms of disease timing, 83 patients presented with synchronous CRLM, while 72 had metachronous CRLM. Preoperative oxaliplatin‐based chemotherapy was administered to 70 of the 155 patients, among whom 25 (35.7%) were found to have BL during intraoperative assessment. The mean evaluation score for EOB‐MRI by two radiologists and the average APRI value were 2.3 ± 0.9 and 4.8 ± 0.2, respectively. When calculating the weighted kappa statistic for the five‐point RLS scale between two radiologists, a high degree of agreement (κ = 0.51, *p* < 0.01) was observed.

Postoperative complications classified as Clavien–Dindo classification ≥ 2 were observed in 22 of the 155 patients (14.2%). These included wound infection (*n* = 9), biloma (*n* = 3), delirium (*n* = 9), pulmonary infection (*n* = 1), ascites (*n* = 9), and deep vein thrombosis (*n* = 6) (Table S1). The postoperative mortality rate following hepatectomy for CRLM was 0%.

### 3.2 Comparison Between BL and Non‐BL Groups

Comparison between the BL group (*n* = 25) and the non‐BL group (*n* = 130) demonstrated that platelet counts were significantly lower, whereas the indocyanine green (ICG) 15‐min values and serum CEA concentrations were significantly higher in the BL group than in the non‐BL group. The APRI was also significantly elevated in the BL group compared with the non‐BL group (5.7 ± 0.4 vs. 4.3 ± 0.2, *p* = 0.0028). Furthermore, the scores obtained from the EOB‐MRI evaluations by radiologists were significantly higher in the BL group than those in the non‐BL group (3.2 ± 0.2 vs. 2.1 ± 0.1, *p* < 0.0001) (Table 1). Patients who received six or more cycles of oxaliplatin‐based chemotherapy exhibited hepatic injury, with the presence of blue liver being documented (Table 1). No significant differences were observed between the two groups in terms of short‐term outcomes, including postoperative complications (*p* = 0.1250) (Table 1).

**TABLE 1:** Comparative analysis of clinicopathological parameters between BL group and non‐BL group.

Variable	non‐BL group (*n* = 130)	BL group (*n* = 25)	*p*
Gender (male/female)	64/66	15/10	0.3239
Age (years)^a^	64.6 ± 1.0	63.8 ± 2.2	0.7524
BMI (kg/m^2^)^a^	23.1 ± 0.3	22.4 ± 0.6	0.3272
Neo‐adjuvant chemotherapy (yes/no)	63/67	25/0	**< 0.0001**
Neo‐adjuvant chemotherapy with oxaliplatin (yes/no)	45/85	25/0	**< 0.0001**
Nubmers of cycles (0–5/≥ 6)	88/42	9/16	**0.0027**
CRC location (right/left/rectum)	36/54/40	1/14/10	0.0537
CRC histology (well/moderate/poor)	2/125/3	3/22/0	0.0696
Esophageal or gastric varices (yes/no)	0/130	0/25	—
Splenomegaly (yes/no)	4/126	2/23	0.2887
Portal hypertension (yes/no)	5/125	2/23	0.3597
Maximum tumor size (cm)^a^	2.6 ± 0.2	2.6 ± 0.4	0.9766
Number of liver metastasis^a^	4.0 ± 0.5	4.8 ± 1.1	0.5075
TBS^a^	5.3 ± 0.5	5.9 ± 1.0	0.5823
Serum albumin (g/dL)^a^	4.0 ± 0.1	3.9 ± 0.1	0.1958
Total bilirubin (mg/dL)^a^	0.7 ± 0.1	0.8 ± 0.1	0.1478
ICG test (%)^a^	7.4 ± 0.3	9.3 ± 0.8	**0.0330**
Platelet (×10^4^/μL)^a^	21.2 ± 0.5	17.4 ± 1.2	**0.0046**
AST (IU/L)^a^	26 ± 1	29 ± 2	0.1167
ALT (IU/L)^a^	25 ± 1	23 ± 3	0.6089
Cr (mg/dL)^a^	0.7 ± 0.1	0.7 ± 0.1	0.4399
CEA (ng/mL)^b^	6 (3.1–29.3)	9.1 (3.6–30.9)	**0.0248**
CA19‐9 (ng/mL)^b^	16.5 (4.3–45.8)	15.0 (8.0–50.0)	0.6124
Distribution (unilobar/bilobar)	77/53	10/15	**0.0236**
Timing of resection (synchronous/metachronous)	68/62	15/10	0.4800
Operative time (min)^a^	254.7 ± 8.6	263.5 ± 19.5	0.6794
Blood loss (g)^a^	221.4 ± 24.6	277.8 ± 61.0	0.3985
Postoperative complication CD (0–1/≥ 2)	114/16	19/6	0.1250
Adjuvant chemotherapy (yes/no)	74/56	15/10	0.7757
APRI score^a^	4.3 ± 0.2	5.7 ± 0.4	**0.0028**
EOB‐MRI evaluation score (points)^a^	2.1 ± 0.1	3.2 ± 0.2	**< 0.0001**

*Note:* Boldface *p* values are statistically significant.

Abbreviations: ALT, alanine aminotransferase; APRI, aspartate aminotransferase to platelet ratio index; AST, aspartate aminotransferase; BL, blue liver; BMI, body mass index; CA19‐9, carbohydrate antigen 19–9; CD, Clavien–Dindo classification; CEA, carcinoembryonic antigen; Cr, creatinine; CRC, colorectal cancer; EOB‐MRI, ethoxybenzyl‐diethylenetriamine pentaacetic acid‐enhanced magnetic resonance imaging; ICG, indocyanine green; TBS, tumor burden score.

^a^ Data are expressed as mean ± standard error.

^b^ Data are expressed as median (25th–75th percentile).

In addition, radiologists assessed preoperative CT scans for imaging findings indicative of collateral circulation and splenomegaly. Preoperative CT revealed features of portal hypertension, including esophageal or gastric varices and splenomegaly, in seven patients, two of whom were found to have blue liver (Table 1).

The survival rate of patients with metastatic liver cancer after hepatectomy was significantly lower in the BL group than that in the non‐BL group (*p* = 0.0338) (Figure S2A). There was no significant difference in DFS between the two groups (Figure S2B).

### 3.3 Comparison Between Reticular Low Signal High and Low Groups by EOB‐MRI

The patients were divided into two groups based on whether they scored ≥ 3 points or < 3 points in the EOB‐MRI evaluation, that is, whether they had RLS or not, and the clinicopathological factors were compared between the two groups. In the high RLS group (*n* = 33, 21.3%), which showed RLS on EOB‐MRI (RLS group), ICG test and serum AST levels were significantly higher than those in the low RLS group (non‐RLS group). Additionally, the incidence of splenomegaly and portal hypertension was significantly higher in the RLS group. During surgery, the RLS group showed a significantly higher incidence of BL and greater blood loss than did the non‐RLS group (Table 2). No significant differences were observed in tumor marker levels, tumor size, differentiation grade, or postoperative complications.

**TABLE 2:** Comparative analysis of clinicopathological parameters between EOB‐MRI evaluation score high and low groups.

Variable	EOB‐MRI evaluation score < 3 points (non‐RLS group) (*n* = 122)	EOB‐MRI evaluation score ≥ 3 points (RLS group) (*n* = 33)	*p*
Gender (male/female)	61/61	18/15	0.6431
Age (years)^a^	65.1 ± 1.0	62.1 ± 1.9	0.1569
BMI (kg/m^2^)^a^	22.8 ± 0.3	23.9 ± 0.5	0.0539
Neo‐adjuvant chemotherapy (yes/no)	62/60	26/7	**0.0040**
Neo‐adjuvant chemotherapy with oxaliplatin (yes/no)	49/73	21/12	**0.0162**
Nubmers of cycles (0–5/≥ 6)	80/42	17/16	0.1387
CRC location (right/left/rectum)	35/54/33	2/14/17	**0.0057**
CRC histology (well/moderate/poor)	3/116/3	2/31/0	0.5827
Esophageal or gastric varices (yes/no)	0/122	0/33	—
Splenomegaly (yes/no)	2/120	4/29	**0.0041**
Portal hypertension (yes/no)	2/120	5/28	**0.0009**
Maximum tumor size (cm)^a^	2.5 ± 0.2	3.0 ± 0.3	0.1876
Number of liver metastasis^a^	4.0 ± 0.5	4.7 ± 0.9	0.4845
TBS^a^	5.2 ± 0.5	6.2 ± 0.9	0.3353
Serum albumin (g/dL)^a^	4.0 ± 0.1	3.9 ± 0.1	0.0632
Total bilirubin (mg/dL)^a^	0.7 ± 0.1	0.7 ± 0.1	0.9251
ICG test (%)^a^	7.4 ± 0.4	8.9 ± 0.7	**0.0408**
Platelet (×10^4^/μL)^a^	20.9 ± 0.6	19.7 ± 1.1	0.3380
AST (IU/L)^a^	25 ± 1	31 ± 2	**0.0045**
ALT (IU/L)^a^	24 ± 1	25 ± 3	0.6237
Cr (mg/dL)^a^	0.7 ± 0.1	0.7 ± 0.1	0.7678
CEA (ng/mL)^b^	6 (2.8–24.3)	11.5 (3.8–42.5)	0.6897
CA19‐9 (ng/mL)^b^	14 (4–41)	19 (8–94.5)	0.8036
Distribution (unilobar/bilobar)	74/48	13/20	0.0767
Timing of resection (synchronous/metachronous)	63/59	20/13	0.3595
Operative time (min)^a^	250.2 ± 8.8	278.0 ± 16.9	0.1467
Blood loss (g)^a^	185.0 ± 26.5	398.8 ± 50.9	**0.0003**
Postoperative complication CD (0–1/≥ 2)	105/17	28/5	0.8589
Adjuvant chemotherapy (yes/no)	67/55	22/11	0.2259
APRI score^a^	4.3 ± 0.2	5.5 ± 0.4	**0.0043**
Blue liver (yes/no)	12/110	13/20	**< 0.0001**

*Note:* Boldface *p* values are statistically significant.

Abbreviations: ALT, alanine aminotransferase; APRI, aspartate aminotransferase to platelet ratio index; AST, aspartate aminotransferase; BMI, body mass index; CA19‐9, carbohydrate antigen 19–9; CD, Clavien–Dindo classification; CEA, carcinoembryonic antigen; Cr, creatinine; CRC, colorectal cancer; EOB‐MRI, ethoxybenzyl‐diethylenetriamine pentaacetic acid‐enhanced magnetic resonance imaging; ICG, indocyanine green; RLS, reticular low signal; TBS, tumor burden score.

^a^ Data are expressed as mean ± standard error.

^b^ Data are expressed as median (25th–75th percentile).

### 3.4 Relationship Between EOB‐MRI Evaluation and APRI Score

We investigated the correlation between EOB‐MRI evaluation and APRI score. The results showed a positive correlation between the EOB‐MRI evaluation and APRI score (*r* = 0.2580, *p* = 0.0012) (Figure S3).

### 3.5 Evaluation of Blue Liver by EOB‐MRI and APRI Scores

When the APRI score was used to distinguish BL using the ROC curve, the cut‐off value was 4.53. When evaluating the group divided into three points based on EOB‐MRI assessment and the group divided based on an APRI score of 4.53, the group with low values in both categories showed BL in 5 (6.3%) of 79 patients. In the group with high EOB‐MRI findings (the RLS group) and low APRI scores, one of 11 patients (9.1%) showed BL. In the group with low EOB‐MRI findings (the non‐RLS group) and high APRI scores, 7 of 43 patients (16.3%) showed BL. In the group with high scores in both categories, 12 of 22 patients (54.5%) showed BL (Table 3).

**TABLE 3:** Blue liver evaluation by both EOB‐MRI imaging and APRI score.

Variable	EOB‐MRI evaluation score < 3 points (non‐RLS group)	EOB‐MRI evaluation score ≥ 3 points (RLS group)
APRI score ≥ 4.53	Blue liver 7 cases (16.3%) in 43 patients	Blue liver 12 cases (54.5%) in 22 patients
APRI score < 4.53	Blue liver 5 cases (6.3%) in 79 patients	Blue liver 1 case (9.1%) in 11 patients

Abbreviations: APRI, aspartate aminotransferase to platelet ratio index; EOB‐MRI, ethoxybenzyl‐diethylenetriamine pentaacetic acid‐enhanced magnetic resonance imaging; RLS, reticular low signal.

### 3.6 ROC Analyses of APRI Score, EOB‐MRI, and a Combination of EOB‐MRI and APRI Scores for BL

When the ROC curve was assessed for BL using the APRI score, EOB‐MRI, and the combination of EOB‐MRI with APRI score, the scoring system in the combination of EOB‐MRI with APRI score was found to be the most accurate in diagnosing BL, with an area under the curve of 0.806, a sensitivity of 78.0%, and a specificity of 67.7% (Table 4). The combination of higher EOB‐MRI and APRI scores significantly increased the diagnostic rate of BL (Table 4).

**TABLE 4:** ROC analyses of APRI score, EOB‐MRI, and a combination of EOB‐MRI and APRI score for blue liver.

Variable	AUC	Cut off	Sensitivity (%)	Specificity (%)
APRI score	0.688	4.53	76.0	64.6
EOB‐MRI	0.752	2.5 (points)	48.0	82.3
A combination of EOB‐MRI and APRI score	**0.806**	—	**78.0**	**67.7**

*Note:* The highest AUC is highlighted in bold values.

Abbreviations: APRI, aspartate aminotransferase to platelet ratio index; AUC, area under the curve; EOB‐MRI, ethoxybenzyl‐diethylenetriamine pentaacetic acid‐enhanced magnetic resonance imaging; ROC, receiver operating characteristic.

### 3.7 Prognosis Based on EOB‐MRI and a Combination of EOB‐MRI and APRI

**FIGURE 3:**
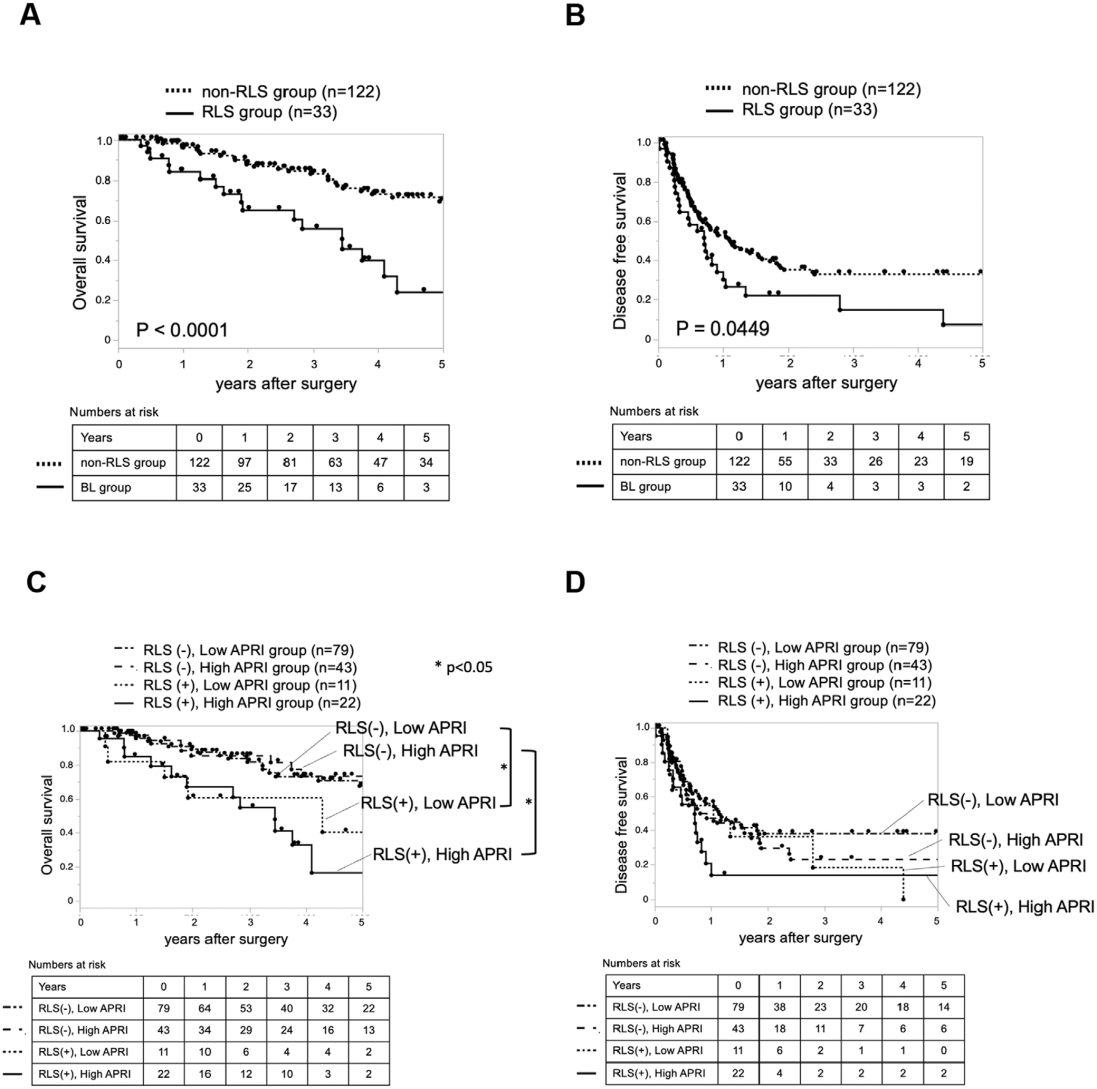
Kaplan–Meier analysis of (A) OS and (B) DFS in patients with CRLM divided into ≥ 3 EOB‐MRI images (RLS group, *n* = 33) and < 3 (non‐RLS group, *n* = 122). Kaplan–Meier analysis of (C) OS and (D) DFS in patients with CRLM for the four groups divided into RLS and non‐RLS groups, and high and low APRI groups. OS, overall survival; DFS, disease‐free survival; CRLM, colorectal cancer liver metastases; EOB‐MRI, ethoxybenzyl‐diethylenetriamine pentaacetic acid‐enhanced magnetic resonance imaging; RLS, reticular low signal; APRI, aspartate aminotransferase to platelet ratio.

When comparing the two groups with ≥ 3 EOB‐MRI images (the RLS group) and < 3 images (the non‐RLS group), the RLS group had significantly worse OS and DFS (Figure 3A,B).

Next, we examined the prognosis based on a combination of two groups of EOB‐MRI images and two groups with high and low APRI scores. When the low APRI group was divided into RLS (+) and RLS (−) groups, the RLS (+) group had a poorer prognosis in terms of OS (*p* = 0.0205, Figure 3C). Similarly, when the high APRI group was divided into RLS (+) and RLS (−) groups, the RLS (+) group had a poorer prognosis in terms of OS (*p* = 0.00026, Figure 3C). No significant differences in DFS were observed among the four groups (Figure 3D).

### 3.8 Univariate and Multivariate Analyses of Clinicopathological Variables in Relation to OS After CRLM Resection

The univariate analysis showed that neo‐adjuvant chemotherapy number of cycles (*p* = 0.045), TBS (*p* = 0.007), and RLS (+) and high APRI (*p* = 0.001) were significantly associated with OS after resection of CRLM (Table 5). The multivariate analysis confirmed that TBS (hazard ratio (HR), 2.20; 95% confidence interval (CI), 1.20–4.05; *p* = 0.011) and RLS (+) and high APRI (HR, 2.25; 95% CI, 1.10–4.58; *p* = 0.026) were independently associated with OS in patients who underwent hepatectomy for CRLM (Table 5).

**TABLE 5:** Univariate and multivariate Cox regression analyses of clinicopathological variables in relation to overall survival after CRLM resection.

Variable	Univariate analysis			Multivariate analysis		
	HR	95% CI	*p*	HR	95% CI	*p*
Gender (male/female)	1.34	0.75–2.41	0.314			
Age (≥ 64.5 vs. < 64.5)	1.07	0.60–1.94	0.813			
BMI (≥ 23.0 vs. < 23.0)	1.14	0.64–2.03	0.647			
Neo‐adjuvant chemotherapy number of cycles (0–5/≥ 6)	1.80	1.01–3.20	**0.045**	1.42	0.79–2.55	0.246
CRC location (colon/rectum)	0.79	0.44–1.42	0.426			
**TBS (≥ 5.4 vs. < 5.4**)	**2.73**	**1.53–4.88**	**0.007**	**2.20**	**1.20–4.05**	**0.011**
Serum albumin (≥ 3.5 vs. < 3.5)	0.42	0.17–1.06	0.067			
ICG test (≥ 7.7 vs. < 7.7)	1.10	0.62–1.95	0.744			
AST (≥ 26 vs. < 26)	1.01	0.56–1.79	0.986			
ALT (≥ 24 vs. < 24)	1.35	0.76–2.39	0.312			
CEA (≥ 6.6 vs. < 6.6)	1.67	0.91–3.15	0.093			
CA19‐9 (≥ 15.0 vs. < 15.0)	1.59	0.88–2.88	0.121			
Distribution (unilobar/bilobar)	0.58	0.32–1.03	0.063			
Timing of resection (synchronous/metachronous)	0.95	0.57–1.69	0.866			
Operative time (≥ 256.1 vs. < 256.1)	1.44	0.81–2.56	0.212			
Blood loss (≥ 230.5 vs. < 230.5)	1.47	0.80–2.72	0.217			
Postoperative complication CD (0–1/≥ 2)	0.64	0.25–1.61	0.338			
Adjuvant chemotherapy (yes/no)	0.67	0.37–1.20	0.180			
**RLS(+) and high APRI (yes/no**)	**3.31**	**1.68–6.53**	**0.001**	**2.25**	**1.10–4.58**	**0.026**

*Note:* Boldface *p*‐values are statistically significant.

Abbreviations: ALT, alanine aminotransferase; APRI, aspartate aminotransferase to platelet ratio index; AST, aspartate aminotransferase; BMI, body mass index; CA19‐9, carbohydrate antigen 19–9; CD, Clavien–Dindo classification; CEA, carcinoembryonic antigen; CI, confidence interval; CRC, colorectal cancer; CRLM, colorectal cancer liver metastases; HR, hazard ratio; ICG, indocyanine green; RLS, reticular low signal; TBS, tumor burden score.

## 4 Discussion

This study demonstrates that a combination of EOB‐MRI and APRI provides a robust, non‐invasive approach for the preoperative diagnosis of SOS, commonly known as “BL,” in patients with CRLM treated with oxaliplatin‐based chemotherapy. Our findings indicate that both EOB‐MRI characteristics and APRI scores are independently and synergistically associated with BL, and that these combined modalities offer significantly improved diagnostic accuracy and prognostic stratification compared with either technique alone.

The clinical significance of SOS as a chemotherapy‐induced liver injury has been increasingly recognized, particularly in the context of preoperative oxaliplatin use [17]. Although oxaliplatin‐based chemotherapy improves the resectability and oncological outcomes of CRLM, it contributes to hepatic vascular injury, resulting in sinusoidal congestion, hepatocyte atrophy, and fibrosis [19]. SOS not only increases the technical difficulty of hepatic resection but also compromises liver function reserve and increases the risk of perioperative complications [20]. Therefore, accurate preoperative assessment of SOS is essential. Despite its clinical relevance, early identification of SOS remains difficult, as histopathological confirmation is not feasible prior to surgery, and conventional imaging lacks standardized diagnostic criteria.

EOB‐MRI provides a visual assessment of hepatocellular uptake and biliary excretion of contrast agents, with the RLS pattern in the hepatobiliary phase representing a surrogate marker for sinusoidal endothelial injury [7]. Our findings show that the RLS on EOB‐MRI correlates strongly with the intraoperative detection of BL, as well as with features of portal hypertension such as splenomegaly and increased intraoperative blood loss. These observations support the utility of EOB‐MRI for visualizing the structural consequences of oxaliplatin‐induced liver injury.

Moreover, although the APRI score is a well‐established surrogate marker of liver fibrosis and inflammation, it can be affected by various factors unrelated to SOS, including viral hepatitis and hematologic disorders [16, 17]. Therefore, the specificity for chemotherapy‐induced SOS should be interpreted with caution. Similarly, although RLS on EOB‐MRI is suggestive of impaired hepatocyte function, it is not pathognomonic for SOS and may overlap with other parenchymal liver diseases. Hence, further validation with larger prospective studies and incorporation of additional non‐invasive markers, such as liver stiffness measurements, may enhance diagnostic precision.

An important finding of this study was the differential incidence of BL in the discordant subgroups defined by the RLS of EOB‐MRI and APRI scores. Specifically, among patients with RLS but with a low APRI score, only 1 of 11 (9.1%) had BL, whereas in the converse group, 7 of 43 patients (16.3%) with non‐RLS but with a high APRI score had BL. These discordant patterns highlight the critical limitations when relying on either imaging or serum biomarker assessment in isolation.

The low BL detection rate in the RLS/low APRI group may be attributed to the limited sensitivity of the APRI in detecting early or localized sinusoidal injury. Although EOB‐MRI reflects morphological and functional hepatocellular changes, including impaired contrast uptake and venous congestion typical of SOS, APRI is primarily a surrogate for systemic sinusoidal injury and fibrosis through indirect markers such as AST elevation and thrombocytopenia. In early‐stage SOS or in patients with focal, patchy involvement, AST levels may remain within the normal range, and the platelet count may not yet decrease, resulting in a falsely low APRI score. Thus, in these patients, MRI may detect local hepatic dysfunction that biochemical markers fail to detect.

Conversely, the low BL detection rate in the non‐RLS/high APRI group suggests that APRI may detect systemic hepatic stress or early portal hypertension before it manifests as morphological abnormalities detectable by EOB‐MRI. EOB‐MRI relies on the functional contrast uptake by hepatocytes, and subtle changes may occur below the radiological detection threshold, particularly in the absence of advanced parenchymal damage. Thus, a high APRI score without corresponding imaging findings may indicate evolving or diffuse SOS that is not visible radiologically. Patients with elevated APRI but low RLS may present with early or diffuse SOS, which could progress over time and exacerbate BL. Therefore, rather than continuing chemotherapy indiscriminately, it may be more appropriate to consider advancing the timing of liver resection once resection becomes feasible. Conversely, patients with low APRI but high RLS may exhibit localized focal SOS. Because blood test results alone may not provide an accurate assessment of hepatic functional reserve, imaging studies should be employed alongside blood tests to evaluate the appropriateness of liver resection and the associated surgical intervention.

These findings collectively underscore the rationale for combining EOB‐MRI and APRI in preoperative evaluation. When both indicators were positive (RLS/high APRI), the probability of BL significantly increased (12 of 22 patients, 54.5%), indicating a high diagnostic yield through multimodal assessment. This synergistic effect reflects the complementary nature of imaging and serologic evaluation; EOB‐MRI captures localized structural and perfusional deficits, and APRI reflects systemic endothelial and hepatocellular injury. In clinical practice, this dual‐modality approach enhances sensitivity and specificity, thereby enabling more accurate risk stratification and surgical planning. Ultimately, the necessity of combining EOB‐MRI and APRI lies in their ability to mitigate each other's limitations and offer a more robust diagnostic framework for detecting SOS. This is especially critical in a perioperative setting, where undetected SOS can lead to increased morbidity, impaired liver regeneration, and worse long‐term outcomes. Identifying individuals at high risk of SOS with the presence of RLS on EOB‐MRI and high APRI might guide surgical decision‐making—such as parenchymal‐sparing approaches or delaying resection to allow hepatic recovery—as well as inform perioperative management strategies aimed at reducing complications.

There were no significant differences between the two groups in terms of tumor biological characteristics, recurrence patterns, or postoperative complications. However, there were significant differences in liver function, including ICG test, AST, and platelet counts (Tables 1 and 2). In our cohort, patients with blue liver had impaired liver function due to oxaliplatin‐induced sinusoidal injury, which may have contributed to poorer tolerance of subsequent treatments, higher susceptibility to non‐cancer‐related complications, and reduced physiological reserve following recurrence. These factors would negatively impact OS, even if the timing or frequency of recurrence (DFS) remained comparable between groups. In other words, although the incidence of recurrence was similar, patients with compromised hepatic reserve were less able to survive after recurrence or withstand subsequent therapies, leading to a discrepancy between OS and DFS outcomes.

This study has several limitations. First, it was retrospective and conducted at a single center, which may have introduced selection and observer bias. Second, although EOB‐MRI offers valuable structural insights, its interpretation may be subject to inter‐observer variability. Although this was mitigated using the average scores from two independent radiologists, future integration of artificial intelligence‐based image analysis may further improve reproducibility. Third, APRI is affected by non‐hepatic factors, including bone marrow suppression and thrombocytopenia due to chemotherapy, potentially limiting its specificity for SOS. Future studies should focus on prospective validation of our findings in larger multicenter cohorts. In addition, longitudinal monitoring of the APRI and imaging changes throughout chemotherapy may enhance the temporal understanding of SOS development and enable earlier intervention. Exploration of additional biomarkers and the incorporation of advanced imaging analytics could further refine diagnostic algorithms.

In conclusion, our study supports the combined use of EOB‐MRI and APRI as a reliable, non‐invasive method for the preoperative diagnosis of SOS in patients with CRLM undergoing hepatic resection after oxaliplatin treatment. This strategy not only improves diagnostic accuracy but also aids in prognostic stratification, thereby contributing to safer surgical planning and better‐informed patient care.

## Author Contributions

**Tomonari Shimagaki:** conceptualization, writing – original draft, data curation, investigation, validation, formal analysis, methodology, project administration. **Keishi Sugimachi:** writing – review and editing, conceptualization, supervision. **Takahiro Tomino:** data curation, formal analysis. **Takeshi Kurihara:** formal analysis, data curation. **Emi Onishi:** data curation, formal analysis. **Yutaro Shimomura:** formal analysis, visualization. **Kenji Shinozaki:** formal analysis, visualization. **Masaru Morita:** writing – review and editing.

## Disclosure

*Animal Studies*: N/A.

## Ethics Statement

The study protocol complied with the ethical guidelines of human clinical research established by the Japanese Ministry of Health, Labor, and Welfare, as well as with the 1964 Helsinki Declaration and its later amendments, and was approved by the Ethics and Indications Committee of the National Hospital Organization Kyushu Cancer Center (No. 2019‐54).

## Consent

Informed consent was obtained from all the participants.

## Conflicts of Interest

The authors declare no conflicts of interest.

## Supporting information


**FIGURE S1:** Intraoperative blue liver findings.

**FIGURE S2:** Kaplan–Meier analysis of (A) OS and (B) DFS in patients with CRLM divided into the BL (*n* = 25) and non‐BL (*n* = 130) groups. OS, overall survival; DFS, disease‐free survival; CRLM, colorectal cancer liver metastasis; BL, blue liver.

**FIGURE S3:** Correlation between EOB‐MRI and APRI scores. EOB‐MRI, ethoxybenzyl‐diethylenetriamine pentaacetic acid‐enhanced magnetic resonance imaging; APRI, aspartate aminotransferase to platelet ratio.

**TABLE S1:** ags370092‐sup‐0004‐TableS1.docx.
