# Supplementary figures and images for: Combination of Ethoxybenzyl‐Diethylenetriamine Pentaacetic Acid‐Enhanced Magnetic Resonance Imaging and a Serum Biomarker Is Useful in the Diagnosis of Hepatic Sinusoidal Disorder After Chemotherapy Treatment
Source: Ann Gastroenterol Surg. 2025 Sep 9;10(2):548–58. doi: 10.1002/ags3.70092 (PMC12962010; doi:10.1002/ags3.70092)

Supplementary Figure 1

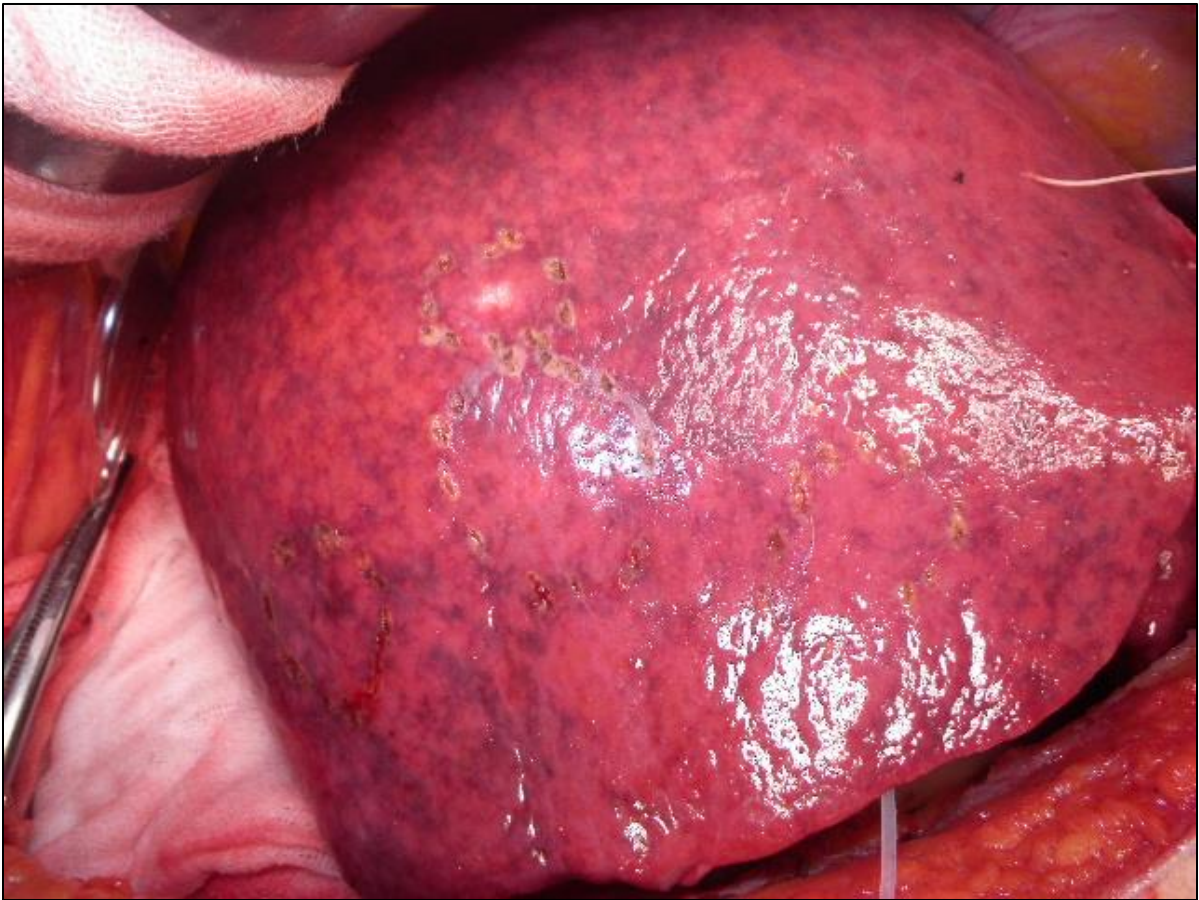

Supplement: Supplementary file 1 — FIGURE S1: Intraoperative blue liver findings. [file AGS3-10-548-s001.pdf]

Supplementary Figure 2

A

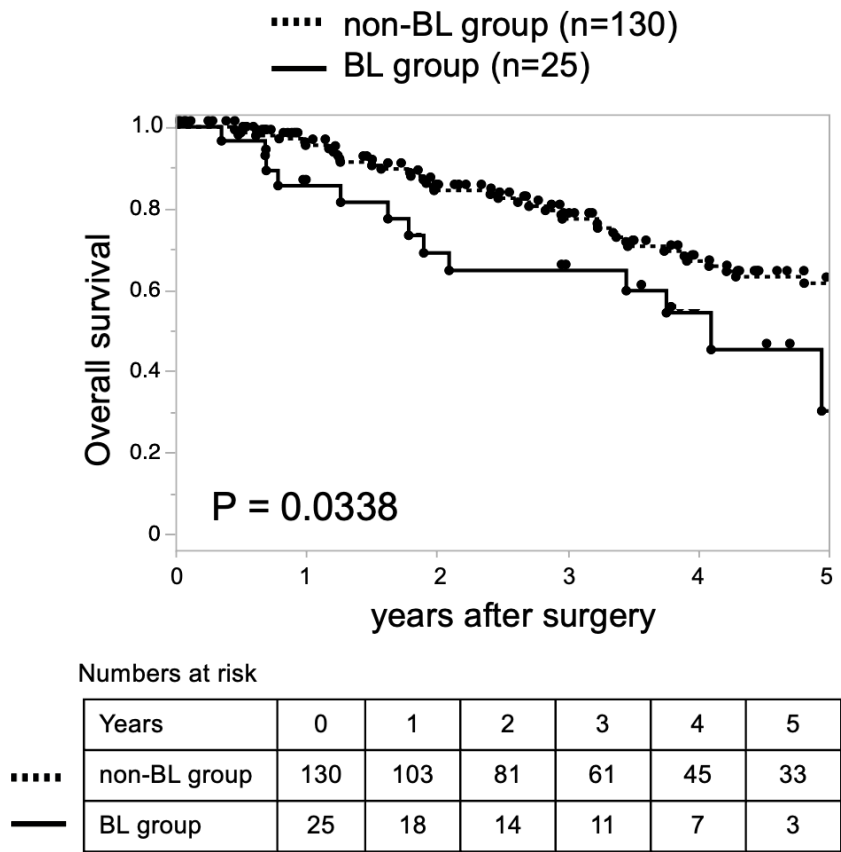

B

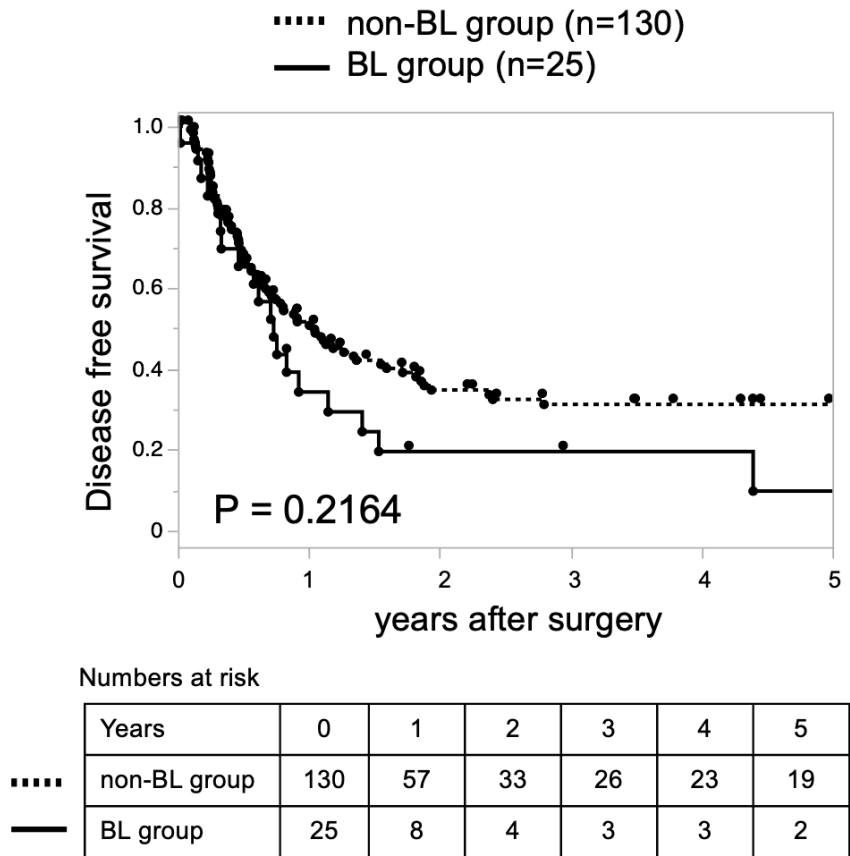

Supplement: Supplementary file 2 — FIGURE S2: Kaplan–Meier analysis of (A) OS and (B) DFS in patients with CRLM divided into the BL (n = 25) and non‐BL (n = 130) groups. OS, overall survival; DFS, disease‐free survival; CRLM, colorectal cancer liver metastasis; BL, blue liver. [file AGS3-10-548-s003.pdf]

Supplementary Figure 3

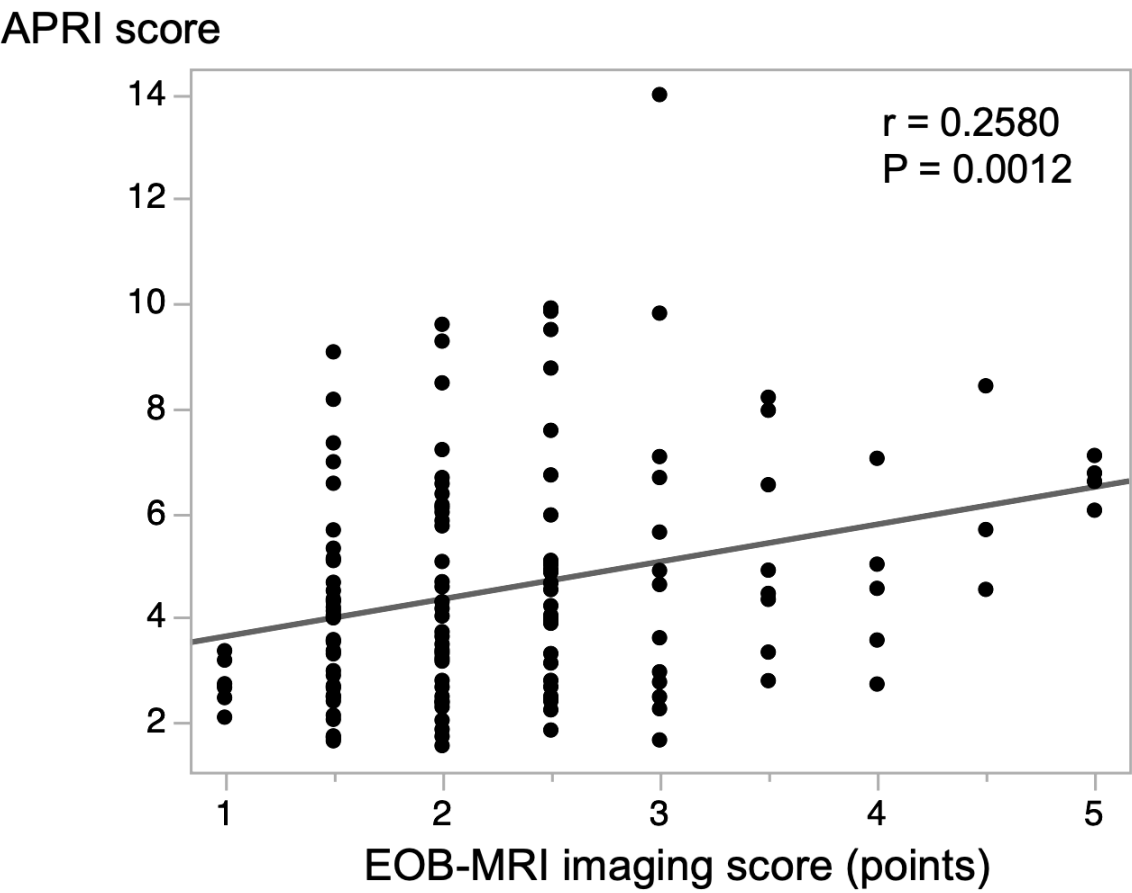

Supplement: Supplementary file 3 — FIGURE S3: Correlation between EOB‐MRI and APRI scores. EOB‐MRI, ethoxybenzyl‐diethylenetriamine pentaacetic acid‐enhanced magnetic resonance imaging; APRI, aspartate aminotransferase to platelet ratio. [file AGS3-10-548-s004.pdf]
