# Supplementary material for: Combination of Ethoxybenzyl‐Diethylenetriamine Pentaacetic Acid‐Enhanced Magnetic Resonance Imaging and a Serum Biomarker Is Useful in the Diagnosis of Hepatic Sinusoidal Disorder After Chemotherapy Treatment
Source: Ann Gastroenterol Surg. 2025 Sep 9;10(2):548–58. doi: 10.1002/ags3.70092 (PMC12962010; doi:10.1002/ags3.70092)
Supplement: Supplementary file 4 — TABLE S1: ags370092‐sup‐0004‐TableS1.docx. [file AGS3-10-548-s002.docx]

**Supplementary Information**

**Combination of ethoxybenzyl-diethylenetriamine pentaacetic acid-enhanced magnetic resonance imaging and a serum biomarker is useful in the diagnosis of hepatic sinusoidal disorder after chemotherapy treatment**

Tomonari Shimagaki^1^, Keishi Sugimachi^1^, Takahiro Tomino^1^, Takeshi Kurihara^1^, Emi Onishi^1^, Yutaro Shimomura^2^, Kenji Shinozaki^2^, Masaru Morita^3^

^1^Department of Hepatobiliary and Pancreatic Surgery, NHO Kyushu Cancer Center, Fukuoka, Japan

^2^Department of Diagnostic Imaging and Nuclear Medicine, NHO Kyushu Cancer Center, Fukuoka, Japan

^3^Department of Gastroenterological Surgery, NHO Kyushu Cancer Center, Fukuoka, Japan

**Supplementary Table 1.** Clinical and tumor characteristics of 155 patients with hepatic colorectal metastases who underwent liver resection

| Variable | n=155 |
| --- | --- |
| Gender (male/ female) | 79/ 76 |
| Age (years)^#^ | 64.5 ± 0.9 |
| BMI (kg/ m^2^)^#^ | 23.0 ± 0.3 |
| Neo-adjuvant chemotherapy  (yes/ no) | 88/ 67 |
| Neo-adjuvant chemotherapy with oxaliplatin (yes/ no) | 70/ 85 |
| Number of cycles (0-5/ ≥6) | 97/ 58 |
| CRC location (right/ left/ rectum) | 37/ 67/ 51 |
| CRC histology  (well/ moderate/ poor) | 3/ 149/ 3 |
| Maximum tumor size (cm)^#^ | 2.6 ± 0.1 |
| Number of liver metastasis^#^ | 4.1 ± 0.4 |
| TBS^#^ | 5.4 ± 0.4 |
| Serum albumin (g/dl)^#^ | 4.0 ± 0.1 |
| Total bilirubin (mg/dl)^#^ | 0.7 ± 0.1 |
| ICG test (%)^#^ | 7.7 ± 0.3 |
| Platelet (×10^4^/µl)^#^ | 20.6 ± 0.5 |
| AST (IU/l)^#^ | 26 ± 1 |
| ALT (IU/l)^#^ | 24 ± 1 |
| Cr (mg/dl)^#^ | 0.7 ± 0.1 |
| CEA (ng/ ml)^##^ | 6.6 (3.2-28.6) |
| CA19-9 (ng/ml)^##^ | 15.0 (5.5-45.5) |
| Distribution (unilobar/ bilobar) | 95/ 60 |
| Timing of resection (synchronous/ metachronous) | 83/ 72 |
| Operative time (min)^#^ | 256.1 ± 7.8 |
| Blood loss (g)^#^ | 230.5 ± 24.5 |
| Postoperative complication CD (0-1/ ≥2) | 133/ 22 |
| Adjuvant chemotherapy (yes/ no) | 89/ 66 |
| APRI score^#^ | 4.8 ± 0.2 |
| EOB-MRI evaluation score (points)^#^ | 2.3 ± 0.9 |

BMI, body mass index; CRC, colorectal cancer; TBS, tumor burden score; ICG, indocyanine green; AST, aspartate aminotransferase; ALT, alanine aminotransferase; Cr, creatinine; CEA, carcinoembryonic antigen; CA19-9, carbohydrate antigen 19-9; CD, Clavien–Dindo classification; APRI, aspartate aminotransferase to platelet ratio index; EOB-MRI, ethoxybenzyl-diethylenetriamine pentaacetic acid-enhanced magnetic resonance imaging.

^#^Data are expressed as mean ± standard error.

^##^Data are expressed as median (25th–75th percentile).
